# Assessing the efficacy of myopia control in monocular orthokeratology treated unilateral myopic children

**DOI:** 10.1186/s12886-022-02693-4

**Published:** 2022-12-19

**Authors:** Yiye Chen, Ce Zheng, Rong Zhu, Lingyan Dong, Jie Cen, Jun Yu, Peiquan Zhao, Xiaoli Kang

**Affiliations:** Department of Ophthalmology, Xin Hua Hospital, Shanghai Jiao Tong University School of Medicine, 1665 KongJiang Road, 200092 Shanghai, China

**Keywords:** Orthokeratology, Myopia control, Efficacy, Axial length

## Abstract

**Purpose:**

To investigate the efficacy of myopia control by comparing the orthokeratology (Ortho-K) treated eyes and the emmetropic contralateral eyes in unilateral myopic children, and to identify the inter-individual influence factors.

**Method:**

In this retrospective study, 1566 medical records of children wearing Ortho-K lens were reviewed, and 62 children who received monocular Ortho-K lens for more than 1 year were analyzed. The change in axial length (AL) of the Ortho-K eyes and the emmetropic contralateral eyes was recorded. To evaluate the absolute and relative efficacy of myopia control, the intra-bilateral absolute reduction in AL growth (ibARAL) and the intra-bilateral relative reduction in AL growth (ibRRAL) were calculated as main outcomes. Association of the AL elongation, ibARAL and ibRRAL with age, sex and ocular parameters was analyzed by correlation analysis and generalized estimating equation (GEE) analysis.

**Result:**

The average initial wearing age was 10.76 ± 1.45 (ranged 8.5 to 15.8). The average baseline SER was − 2.15 ± 1.03 (ranged − 5.25 to -1.00) D in the Ortho-K eyes and − 0.01 ± 0.40 (ranged − 0.75 to 0.75) D in the contralateral eyes. At the 1-year follow-up, the average increased AL was significantly less in the Ortho-K eyes (0.07 ± 0.18 mm) than in the fellow eyes (0.48 ± 0.24 mm) (*p* < 0.001). The mean ibARAL was 0.41 ± 0.30 mm, and the mean ibRRAL was 83.4%±56.3%. In the GEE model, the AL change in Ortho-K eyes (β = 0.051, *p* = 0.009, 95%CI: 0.012 to 0.090), the ibARAL (β= -0.153, *p* = 0.000, 95%CI: -0.228 to -0.078) and the ibRRAL (β= -0.196, *p* = 0.020, 95%CI: -0.361 to -0.030) were independently associated with the spherical equivalent refraction (SER) of the Ortho-K eyes, after adjusting for age, sex, and keratometry.

**Conclusion:**

In our study, the Ortho-K treatment was efficacious in controlling axial length growth in the monocular orthokeratology treated unilateral myopic eyes. The efficacy increased when the myopia was more severe. In the children from 8 to 16 years old, the effectiveness was independent of age and sex.

**Supplementary Information:**

The online version contains supplementary material available at 10.1186/s12886-022-02693-4.

## Introduction

Myopia is currently the most common type of refractive error and has become a worldwide public health issue, especially in East Asia [1, 2]. Moreover, due to heavy near work and academic pressure, the myopic population has become increasingly younger in China [3]. Early-onset myopia without appropriate treatment are more likely to progress to an extremely high degree, which can cause a series of ocular complications, such as cataract, glaucoma and retinal complications, and increases the risk of severe and irreversible vision loss [4, 5].

Various methods have been implemented to control myopia progression, including atropine eye drops, multifocal contact lenses, defocus incorporated spectacles, and orthokeratology (Ortho-K) [6–8]. Among these options, Ortho-K has proved to be an effective treatment option to control myopia in children [8].

Several studies were focused on the efficacy of Ortho-K for myopia control, since the effectiveness is the most concerning issue of parents and physicians. Clinically, myopia progression can be estimated by axial length (AL) elongation. Previous studies showed the results of Ortho-K slowing AL elongation by 32 to 55% [7, 9–11]. The relative value was calculated from the reduction in the mean AL elongation in the Ortho-K treated group compared to the control single vision group. However, heterogeneity of subjects, such as genetic background, levels of myopia, age, progression rate, race, environment, behavior habits, and the duration of follow-up, may influence the results deviated from individual response to treatment [12]. Studies in anisometropic myopic children setting untreated fellow eyes as control may eliminate these factors.

There were a few studies reporting the effect of Ortho-K in anisometropic myopic eyes, and demonstrated that monocular Ortho-K was effective on suppression of AL elongation in the myopic eyes and reduced anisometropia in unilateral myopic children [12–15]. However, the efficacy of myopia control in the unilateral myopic children has not been clarified. This study aimed to investigate the individual absolute and relative efficacy of myopia control by comparing the absolute and relative inter-eye differences of AL elongation between Ortho-K treated eyes and untreated emmetropic fellow eyes in the first year of wearing Ortho-K lens, and to analyze the potential influence factors that may be related to different AL control rate.

## Methods

### Subjects

In this retrospective cohort study, we reviewed 1566 medical records of myopic children who underwent orthokeratology treatment at Xin Hua Hospital affiliated to Shanghai Jiao Tong University School of Medicine (Shanghai, China) from January 2014 to December 2019. Among all, 98 patients with unilateral myopia who received monocular Ortho-K lens treatment were identified.

The inclusion criteria in this study were as follows: over 8 years old at the first visit; cycloplegic spherical equivalent refraction (SER) of-1.00 D or more in the treated eyes; cycloplegic SER between − 0.75 D and + 0.75 D in the contralateral emmetropic eyes; no history of using medications or optical corrections that might affect refractive results. We excluded subjects with the following exclusion criteria: other ocular diseases (including strabismus or ocular disorders), and any systemic or developmental problems that might affect refractive development.

**Fig. 1:**
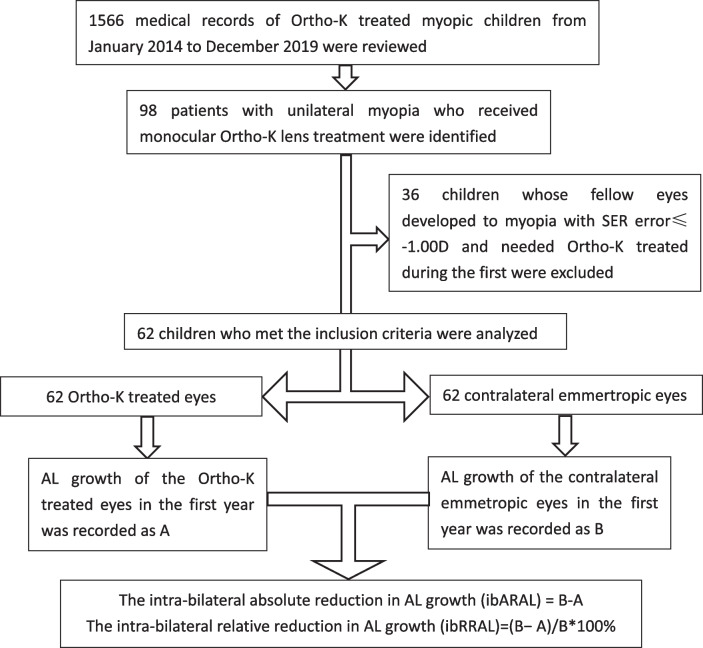
Flow chart of the study

Among the 98 monocular Ortho-K lens treated children, we further excluded 36 children whose fellow eyes developed to myopia with SER error≤-1.00D and needed Ortho-K treated during the first year. Finally, data of 62 children who met the inclusion criteria were analyzed (Fig. 1).

### Materials

The Ortho-K lenses used in this study were Lucid lenses (Lucid Korea Co.,Ltd, Gyeongsangbuk-do, Korea). They were made of Boston XO DK100 with the following physical characteristics: oxygen permeability coefficient, > 90 × 10 − 11 (cm2 *mlO2)/(s*ml*mmHg); diameter, 10.0–11.0 mm, and optical center thickness, 0.23 mm.

Lens fitting was performed according to the manufacturer’s instructions based on corneal topography (Pentacam HR, Oculus, Wetzlar, Germany), cycloplegic refraction, and the horizontal visible iris diameter. Fluorescein was used to observe the centration and tightness of the Ortho-K lens. The final Ortho-K prescriptions were determined by subjective refraction on children who had worn the appropriate trial Ortho-K lenses. The same practitioner fitted all Ortho-K lenses using standardized fitting criteria. All patients were advised to wear their Ortho-K lenses every night for at least eight consecutive hours, except when they had ocular or systemic complication and were advised by doctor to suspend wearing.

### Measurements and follow-up

All patients were required to attend routine Ortho-K aftercare visits (1 day, one week, one month, and every three months after lens delivery) and unscheduled visits when necessary. At each visit, visual acuity was measured using a Snellen chart, and slit-lamp examinations were performed to monitor corneal health and Ortho-K lens integrity. The AL of the eyeball was measured using IOL Master (IOL Master; Zeiss, Jena, Germany). The mean value of five measurements was recorded.

Two main outcomes were obtained and used as indicators of treatment effect: the intra-bilateral absolute reduction in AL growth (ibARAL) and the intra-bilateral relative reduction in AL growth (ibRRAL). Their definition could be referred to as Eqs. (1) and (2) listed below:

1 $$\mathrm{ibARAL}=\mathrm B-\mathrm A$$

2 $$\mathrm{ibRRAL}=(\mathrm B\:-\:\mathrm A)/\mathrm B\ast100\%$$

A: AL growth of the Ortho-K treated eye, B: AL growth of the untreated fellow eye.

### Analysis and statistics

All analyses were performed with SPSS version 19.0 software (IBM Corp., Armonk, New York). Changes in AL from baseline to after one year of treatment were evaluated and compared between the Ortho-K treated myopic eyes and the contralateral emmetropic eyes. Continuous variables of baseline characteristics were expressed as mean ± standard deviation (SD) and evaluated by paired t-test. Categorical variables, such as sex and side of the Ortho-K treated eye were expressed as percentages (%). The changes in AL, ibARAL and ibRRAL were analyzed their correlation with the age, sex, and ocular parameters by correlation analysis. A generalized estimating equation (GEE) model was used to evaluate the associations between potential factors and the difference of AL growth. A value of *P* < 0.05 was considered statistically significant.

## Results

A total number of 62 children (27 males and 35 females) with unilateral myopia were analyzed for the first year of wearing Ortho-K lens. The average initial wearing age was 10.76 ± 1.45 (ranged 8.5 to 15.8). 65% of the Ortho-K treated eyes are right eyes. The average SER was − 2.15 ± 1.03 (ranged − 5.25 to -1.00) D in the Ortho-K wearing eyes and − 0.01 ± 0.40 (ranged − 0.75 to 0.75) D in the contralateral eyes (*p* < 0.001). There is no statistical difference in the initial flat keratometry (FK), steep keratometry (SK), or initial best-corrected visual acuity between the two groups of eyes. Corneal astigmatism was slightly less in the Ortho-K eyes (*p* = 0.030). The detailed demographic and baseline data are described in Table 1.

**Table 1:** Demographic and baseline data

	OK eyes (*n* = 62)	Contralateral eyes (*n* = 62)	*p*-value^△^
Age, Y	10.76 ± 1.45 (ranged 8.5 to 15.8)	10.76 ± 1.45 (ranged 8.5 to 15.8)	
Sex	35 (56%) Female, 27 (44%) Male	35 (56%) Female, 27 (44%) Male	
Eye side	65% Right, 35% Left	35%Right, 65% Left	
FK, D	42.95 ± 1.25	42.91 ± 1.33	0.349
SK, D	43.92 ± 1.41	43.98 ± 1.53	0.299
Corneal astigmatism, D	0.98 ± 0.36	1.08 ± 0.47	0.029*
SER, D	-2.15 ± 1.03	-0.01 ± 0.40	0.000**

*OK* Ortho-k treated, *FK* flat keratometry, *SK* steep keratometry, *SER* cycloplegic spherical equivalent refraction

^△^Paired t-test; *p < 0.05, **p < 0.01

The average baseline AL was significantly longer in the Ortho-K treated eyes (24.41 ± 0.81 mm) than in the contralateral eyes (23.52 ± 0.69 mm) (*p* < 0.001). After 1-year follow-up, the average increased AL was significantly less in the Ortho-K eyes (0.07 ± 0.18 mm) than in the fellow eyes (0.48 ± 0.24 mm) (*p* < 0.001). The mean ibARAL was 0.41 ± 0.30 mm, and the mean ibRRAL was 83.4%±56.3% (Table 2). The ibARALs and ibRRALs were positive in 60 of 62 (96.8%) children, which meant the change in AL in Ortho-K eyes is less than in the fellow eyes in almost all the subjects.

**Table 2:** Data of axial length

	OK eyes (*n* = 62)	Contralateral eyes (*n* = 62)	*p*-value^△^
Baseline AL, mm	24.41 ± 0.81	23.52 ± 0.69	0.000**
ΔAL, mm	0.07 ± 0.18	0.48 ± 0.24	0.000**
ibARAL, mm	0.41 ± 0.30		
ibRRAL	83.4%±56.3%		

*OK* Ortho-k treated, *AL* axial length, *ΔAL* change in axial length, *ibARAL* intra-bilateral absolute reduction in axial length, *ibRRAL* intra-bilateral relative reduction in axial length

^△^Paired t-test; **p < 0.01

**Fig. 2:**
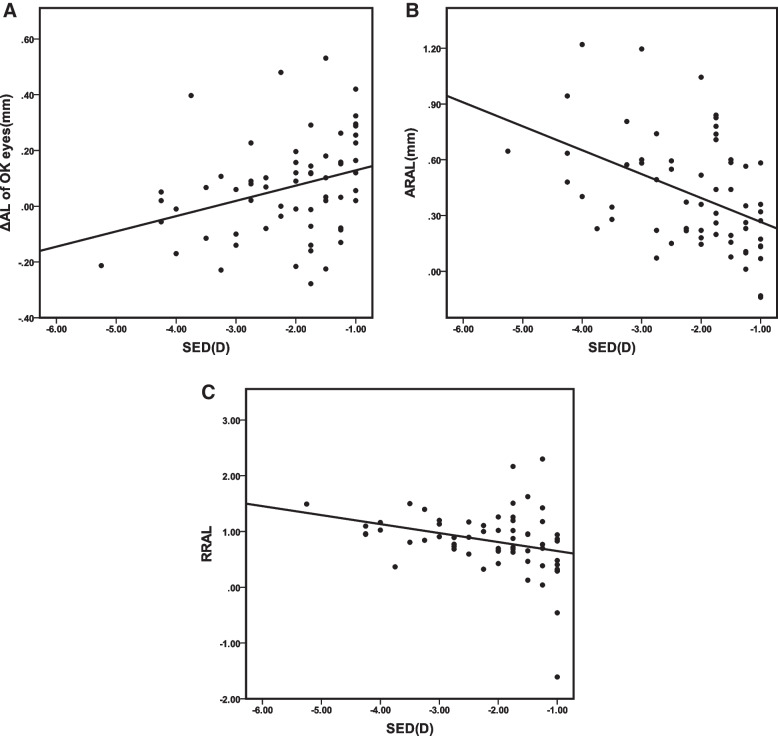
Scatter plots showing the linear correlation between SER and the axial change in Ortho-K treated eyes (**A**), ibARAL (**B**), ibRRAL (**C**). ΔAL: change in axial length; ibARAL: intra-bilateral absolute reduction in axial length; ibRRAL: intra-bilateral relative reduction in axial length; OK: Otho-k treated; SER: cycloplegic spherical equivalent refraction

In univariate analysis, age, sex and SER showed significant correlation with the AL change in Ortho-K treated eyes (all *p* < 0.05), and only SER showed a significant correlation with the ibARAL (*R*=-0.444, *p* < 0.001), and the ibRRAL (*R*=-0.297, *p* = 0.019), respectively (Table 3; Fig. 2).

**Table 3:** Association of ΔAL, ibARAL and ibRRAL with age, sex and ocular parameters

	ΔAL of OK eyes, mm		ΔAL of fellow eyes, mm		ibARAL, mm		ibRRAL	
	R^△^	*p*-value	R^△^	*p*-value	R^△^	*p*-value	R^△^	*p*-value
Age, Y	-0.250	0.049*	-0.070	0.426	0.044	0.732	0.013	0.918
Sex (male versus female)	0.214^+^	0.043*	0.055^+^	0.599	-0.055^+^	0.604	-0.155^+^	0.142
SER, D	0.318	0.012*	0.022	0.818	-0.444	0.000**	-0.297	0.019*
FK, D	-0.029	0.820	0.038	0.670	0.062	0.633	0.033	0.799
SK, D	-0.049	0.706	0.051	0.563	0.090	0.485	0.030	0.816
SK-FK, D	-0.088	0.494	0.007	0.941	0.138	0.286	0.004	0.978
Base AL	-0.073	0.574	-0.047	0.593	0.103	0.424	0.095	0.462

*ΔAL* the change in axial length, *ibARAL* the absolute reduction in axial length, *ibRRAL* the relative reduction in axial length, *OK* Ortho-k treated, *SER* cycloplegic spherical equivalent refraction, *SK* steep keratometry, *FK*: flat keratometry

^△^Pearson correlation coefficient; ^+^Kendall correlation coefficient; *p < 0.05, **p < 0.01

In the GEE model, the AL change in Ortho-K eyes (β = 0.051, *p* = 0.009, 95%CI: 0.012 to 0.090), the ibARAL (β= -0.153, *p* = 0.000, 95%CI: -0.228 to -0.078) and the ibRRAL (β= -0.196, *p* = 0.020, 95%CI: -0.361 to -0.030) were independently associated with the spherical equivalent refraction (SER) of the Ortho-K eyes, after adjusting for age, sex, and keratometry (Table 4).

None of the eyes developed infectious keratitis or conjunctivitis. At routine follow-up examinations, corneal staining of grade 1 or 2 was present in 11 (17.7%) OK eyes at routine follow-up examinations. These recovered completely after discontinuation of lens wear for 3 days to 1 week. No other severe complications, such as corneal ulcer, were noted in the OK eyes and there were no adverse events in the contralateral eyes.

**Table 4:** GEE model for factors associated withΔAL of OK eyes, ibARAL and ibRRAL

	ΔAL of OK eyes, mm			ibARAL, mm			ibRRAL		
	β	95% CI	*p*-value	β	95% CI	*p*-value	β	95% CI	*p*-value
Age, Y	-0.012	-0.040 to 0.016	0.399	-0.034	-0.092 to 0.024	0.251	-0.064	-0.204 to 0.077	0.374
Sex	-0.100	-0.194 to -0.006	0.038*	0.073	-0.070 to 0.216	0.318	0.213	-0.130 to 0.556	0.224
SER, D	0.051	0.012 to 0.090	0.009**	-0.153	-0.228 to -0.078	0.000**	-0.196	-0.361 to -0.030	0.020*
FK , D	0.075	-0.071 to 0.221	0.314	-0.118	-0.365 to 0.129	0.349	-0.004	-0.440 to 0.432	0.985
SK , D	-0.062	-0.196 to 0.073	0.368	0.127	-0.091 to 0.346	0.253	0.007	-0.364 to 0.377	0.972

*ΔAL* change in axial length, *OK* Ortho-K treated, *ibARAL* intra-bilateral absolute reduction in axial length, *ibRRAL* intra-bilateral relative reduction in axial length, *SER* cycloplegic spherical equivalent refraction, *SK* steep keratometry; FK: flat keratometry

*p < 0.05, **p < 0.01

## Discussion

Orthokeratology has been proved to be an effective intervention to slow down myopia progression in children [7–11]. However, the efficacy of myopic control with Ortho-K treatment varied in previous studies. Most of the studies were carried out with one group of Ortho-K treated subjects and one control group of separate subjects wearing conventional spectacles, rigid-gas permeable (RGP) lenses, or single-vision soft contact lenses. Although the difference between groups could be minimized via careful match of age, sex, refractive status, and race, it is still hard to eliminate the difference in individual genetic background, behavioral habit, and environmental visual stimulations. In addition, inevitable dropouts might confound subject matching between the study and control groups and have implications for the clinical evaluation of myopia control.

The emmetropic contralateral eye without any medical treatment was supposed to be the best natural control because it can avoid the impact of between-subject AL difference and the natural growth of AL over time; thus the AL difference is directly related to the impact of Ortho-K treatment [12]. Previously, a few studies focused on the contralateral comparison in unilateral myopic children wearing monocular Ortho-K lens, and demonstrated that Ortho-K treatment could retard the AL growth of myopic eyes and reduce anisomyopic values [12–15]. In our current report, we set the contralateral untreated eye as control, and introduced the ibARAL and the ibRRAL as main outcome for the first time, to represent the individual absolute efficacy and relative efficacy of myopia control compared with the contralateral fellow eye, and then investigate the inter-individual influence factors relevant to the efficacy.

In previous studies that setting spectacles-wearing subjects as control groups, the mean of annual AL elongation varied from − 0.05 to 0.20 mm in Ortho-K treated eyes, and ranged from 0.09 to 0.37 mm in control eyes; thus wearing Ortho-K lenses could induce 32 to 56% reduction in myopic progression [7–11, 16–20]. In our fellow eye control study, the AL elongation of OK eyes was comparable to that in aforementioned studies, while the contralateral control eyes showed visible faster AL growth than control eyes in those studies. As a result, the averaged ibRRAL was as high as 83%, and the averaged ibARAL reached up to 0.41 mm in our study, so we showed a much better result of myopic control by orthokeratology than above-mentioned studies. It is not clearly identified yet that whether one eye wearing OK eyes might have a potential effect on the contralateral untreated eye and accelerate its AL growth. Meanwhile, it is unclear whether the contralateral emmetropic eyes in anisometropic myopes behave the same as bilateral myopes. In the study of unilateral myopic children carried by Long W [15] et al., the annual AL elongation of OK eyes was 0.05 ± 0.19 mm and that of non-myopic eyes was 0.34 ± 0.21 mm in the unilateral OK lens wearing group, while in the spectacle wearing group, the annual AL elongation was 0.33 ± 0.29 mm for the myopic and 0.31 ± 0.32 mm for the non-myopic eyes, which showed that AL elongation in the non-myopic eyes were comparable between unilateral OK group and spectacle group. Wei-Shan Tsai et al. [12] also compared the inter-eye difference of the axial elongation in unilateral myopic children wearing monocular Ortho-K lens. In that study, the AL growth of the two eyes was not shown, but the inter-eye difference of the AL changed from 0.83 mm to 0.68 mm after one year, which means the 1-year ibARAL is 0.15 mm in that study and is much less when in comparison with our results. We speculated that the reason accounting for the difference was that children in our study were from a metropolitan area of China, where the student’s academic burden was heavy, near-work was busy and outdoor activity was insufficient. So the AL elongation was more rapid without any treatment, and the reduction in AL growth was more significant when treated by Ortho-K lens. Besides, different design of Ortho-K lens may induce different area treatment zone and different effect of AL control. This should be taken into consideration of the relative better results compared with other studies.

The baseline SER of the OK eyes is the only factor that showed a significant correlation with the absolute reduction and the relative reduction in our study. In previous studies, Cho et al. [20] found that the eye elongation is faster in those with lower baseline myopia wearing Ortho-K. Hiraoka et al. [7] also found a positive relationship between the AL progression and the baseline myopia in subjects with Ortho-K treatment. Whereas the results of more recent studies carried out by Cho and Cheung [10], Chen et al. [9], and Santodomingo-Rubido et al. [21] showed that axial elongation was not significantly correlated to initial myopia. Our results of correlation analysis demonstrated that the annual AL elongation of OK eyes declined, and the absolute and the relative reduction in AL growth increased significantly when the baseline myopia of OK eyes became more severe. According to previous peripheral myopic defocus hypothesis [22, 23], not only correcting central refractive error but also creating peripheral defocus may be the mechanism of myopia control with the OK lens. It seems to be a reasonable assumption that when more severe myopia is corrected by Ortho-K, a greater decrease of hyperopic defocus is induced in the peripheral retina, thereby exerting a greater suppressive effect on axial growth.

In this study, the AL growth of the OK eyes was statistically significantly smaller in females than in males, while there was no difference in the AL growth of the contralateral eyes between males and females. This phenomenon is consistent with the study by Santodomingo-Rubido et al. [19], even though there is no supportive explanation for it currently. Lesser AL growth of girls wearing OK lens might be partly explained by the carefulness and compliance of most females. However, the undifferentiated ibARAL as well as ibRRAL in both sex groups also demonstrated that OK lens benefits myopic control in either sex.

The univariable analysis showed a correlation between axial elongation of OK eyes and the initial age of the subjects. But in the GEE model, after adjusting for other potential relevant factors, none of the axial elongation, ibARAL or ibRRAL was associated with the initial age. There were also contradictory findings in previous studies. Some studies demonstrated that myopic progression in children had a negative correlation with age [24–26], some reported that younger children might benefit more from wearing OK lens [9, 10], while some studies show no significant correlation between the change in AL and initial age of OK lens [27, 28]. Our sample may not have been powered to identify age interaction effects, which explains the discrepancy between the current and previous findings. And the retrospective nature of our study may also limit the investigation of correlation between age and change in AL.

Two eyes are simultaneously designed as subjects in observed and control groups in this study. This inter-eyes self-contrasted method could reduce the required subject numbers while maintaining statistical power and minimizing the impact of individual differences such as genetic and environmental factors [12, 29, 30]. As a result, these findings had higher sensitivity and reliability.

There are some limitations to the present study. Firstly, the current retrospective study only involved a relatively small study population with a short follow-up period. It is proposed to conduct a further investigation either in a larger sample size or in a longer follow-up period, or both to verify that. However, it is unlikely to keep contralateral eyes emmetropia without optical correction for several years. Rigid gas permeable contact lens may be introduced to the control eyes. Secondly, although contralateral emmetropic eyes were set as a control group, it would be more precise to use a contact lens without any treatment effect for those eyes, but it probably causes concern and disagreement from patients. Thirdly, the diameter of pupil and the area of the central vision correction zone were not recorded in our study, which might have influence on the effect of myopia control. Furthermore, the difference in the baseline AL of bilateral eyes may have influence on the growth of AL in the Ortho-K treated eyes and in the control eyes. Although we had excluded subjects with hyperopia fellow eye in the study to minimize this effect, the limitation of this study design should be considered.

## Conclusion

In conclusion, this study demonstrated the Ortho-K treatment to be efficacious in controlling axial length growth in the monocular orthokeratology treated unilateral myopic eyes. The efficacy increased when the myopia was more severe. Meanwhile, in the children from 8 to 16 years old, the effectiveness was independent of age and sex. Future prospective study on unilateral myopia with Ortho-K treatment may further confirm the findings in this study.

## Supplementary Information

**Additional file 1.**

## Data Availability

All data generated or analysed during this study are included in this published article [data.xlsx].
